# Peripancreatic Fluid Collection Complicated by Endoscopic Ultrasound-Guided Fine-Needle Aspiration

**DOI:** 10.14309/crj.0000000000000432

**Published:** 2020-07-23

**Authors:** Tsuyoshi Suda, Kazuya Kitamura, Shuichi Kaneko

**Affiliations:** 1 Department of Gastroenterology, Kanazawa University Hospital, Kanazawa, Ishikawa, Japan

## CASE REPORT

**Figure 1.**
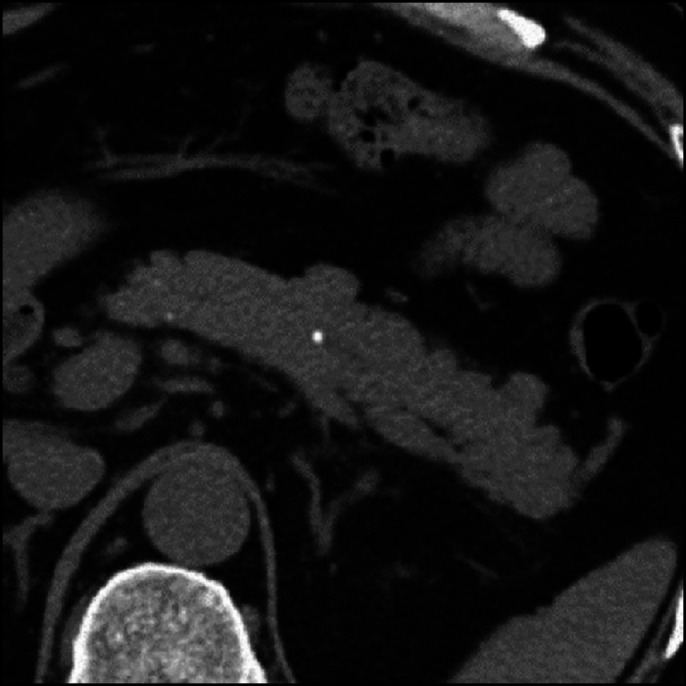
Small pancreatic calculus on computed tomography.

A 54-year-old man was admitted for evaluation of a small pancreatic calculus that was detected incidentally by computed tomography (CT) (Figure 1). Endoscopic ultrasound (EUS) revealed an impacted pancreatic calculus measuring 3.4 mm in the pancreatic body, causing dilatation of the pancreatic duct, with evidence of early chronic pancreatitis; hyperechoic foci, lobular contour, and hyperechoic margins in the main pancreatic duct (MPD). Because serum immunoglobulin G4 level was elevated (154 mg/dL), EUS-guided fine-needle aspiration (EUS-FNA) was planned for histological diagnosis of autoimmune pancreatitis.

**Figure 2.**
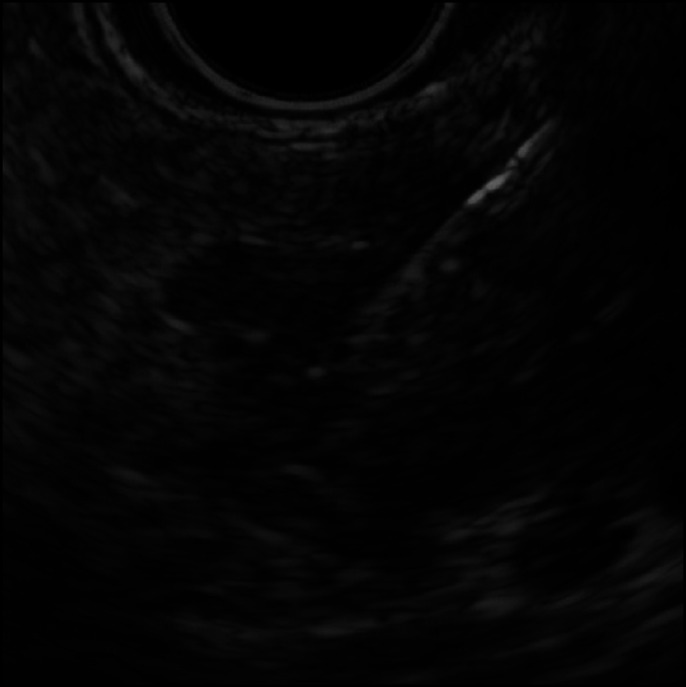
Endoscopic ultrasound-guided fine-needle aspiration performed only by one pass with a 19-gauge needle while carefully avoiding puncture of the pancreatic duct.

EUS-FNA was performed via a transgastric approach with EUS (GF-UCT240; Olympus Medical Systems, Tokyo, Japan). One pass was made with a 19-gauge needle (Sono Tip Pro Control; Medico's Hirata, Osaka, Japan) at the cranial end of the calculus and monitored to avoid puncture of the pancreatic duct (Figure 2). Pathological findings revealed an almost normal pancreatic tissue without evidence of autoimmune pancreatitis.

The next day, the patient complained of epigastric pain. Blood tests revealed remarkably elevated pancreatic enzyme levels (serum amylase: 1229 IU/L). Dynamic CT showed no evidence of typical acute pancreatitis such as focal or diffuse parenchymal enlargement and changes in density due to edema. Concerning the MPD, no dilation was observed, and CT confirmed its continuity. Thus, we considered that a branch duct of the pancreas was injured occasionally by EUS-FNA, resulting in this condition with semblance to a disrupted duct syndrome.

**Figure 3.**
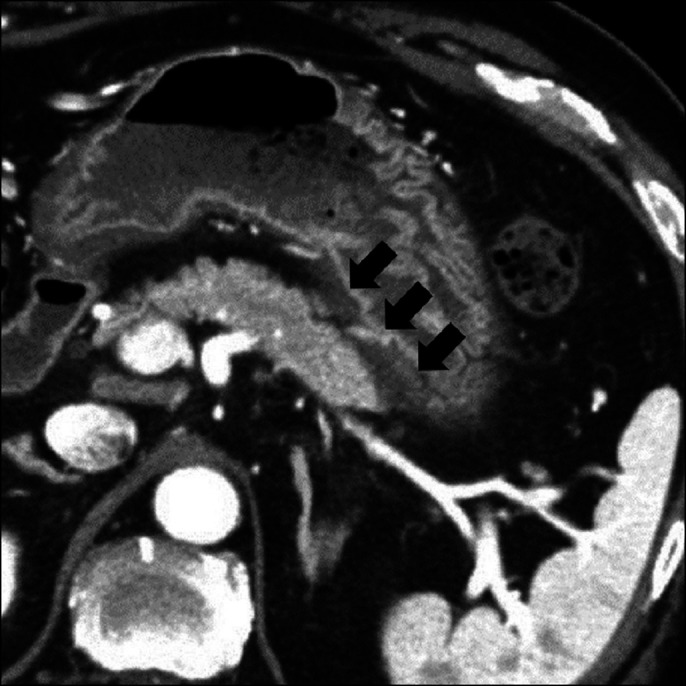
Minimal fluid between the gastric wall and pancreatic body (black arrows) detected around the fine-needle aspiration route on dynamic computed tomography. The black arrows point out minimal fluid correction.

**Figure 4.**
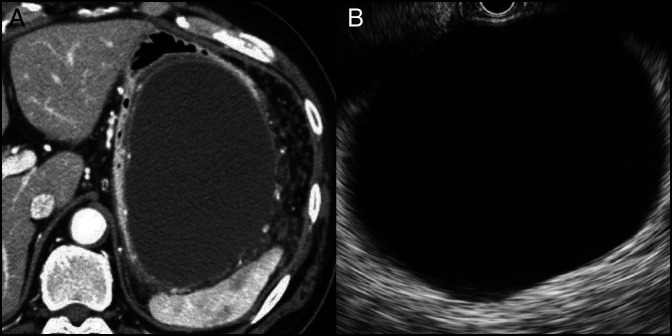
Large pancreatic pseudocyst with severe gastric compression on (A) dynamic computed tomography and (B) endoscopic ultrasound.

However, minimal fluid between the gastric wall and pancreatic body was detected around the FNA route (Figure 3). Epigastric pain and elevated pancreatic enzyme levels persisted. Dynamic CT was repeated 3 days after EUS-FNA, and a pancreatic pseudocyst was detected. Conservative treatment was unsuccessful. The pancreatic pseudocyst enlarged rapidly (>10 cm), with severe gastric compression (Figure 4).

**Figure 5.**
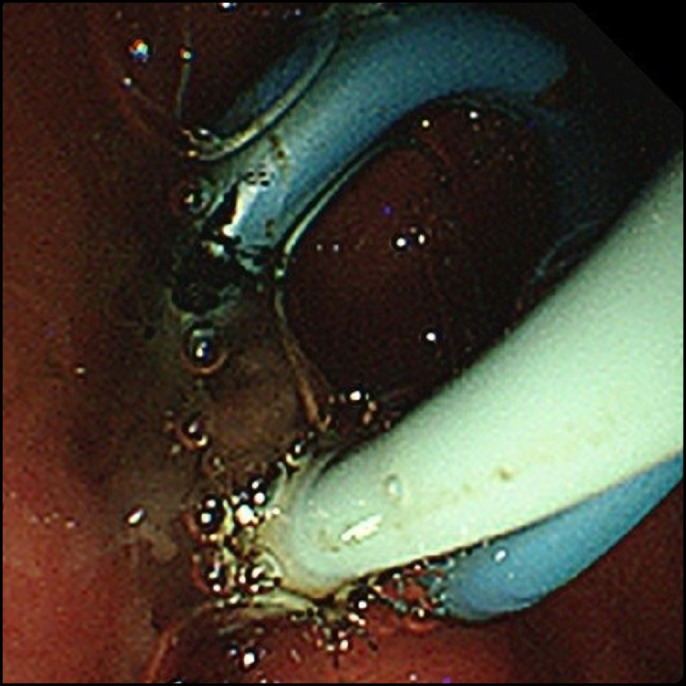
Endoscopic ultrasound-guided pancreatic pseudocyst drainage, with successful internal and external drainages.

Thus, we decided to perform EUS-guided pancreatic cyst drainage (Figure 5). Eventually, after drainage, the pancreatic cyst reduced, and the patient recovered and was discharged. No recurrence was observed after EUS-guided pancreatic cyst drainage; complete resolution was achieved. Although endoscopic retrograde cholangiopancreatography could not be performed to evaluate the MPD after improvement because the patient did not consent to it, dynamic CT and magnetic resonance cholangiopancreatography showed apparent disconnection, at least in the MPD.

EUS-FNA is an established technique with few complications. The overall risk of complications is relatively low (1.2%–1.6%), with no severe or fatal incidences.^1,2^ No pancreatic pseudocyst complicated by EUS-FNA has been described in previous multicase studies.^1–3^ To our knowledge, pancreatic pseudocysts associated with EUS-FNA are extremely rare, with only 2 previous reports.^4,5^ Pancreatic duct injury secondary to FNA was considered a possible cause.^4^ There was no significant difference in complications between using a 19-gauge needle and using other needles sizes in the previous reports.^1,2^ However, we believe that using a thick 19-gauge needle may have been a risk factor in our case.

## DISCLOSURES

Author contributions: T. Suda edited the manuscript, reviewed the literature, and is the article guarantor. K. Kitamura edited the manuscript. S. Kaneko edited the manuscript and provided expert opinions about gastroenterology.

Financial disclosure: None to report.

Informed consent was obtained for this case report.
